# Nanoscale heterogeneity of ultrafast many-body carrier dynamics in triple cation perovskites

**DOI:** 10.1038/s41467-022-33935-0

**Published:** 2022-11-03

**Authors:** Jun Nishida, Peter T. S. Chang, Jiselle Y. Ye, Prachi Sharma, Dylan M. Wharton, Samuel C. Johnson, Sean E. Shaheen, Markus B. Raschke

**Affiliations:** 1 Department of Physics, University of Colorado, Boulder, CO 80309 USA; 2 JILA, University of Colorado, Boulder, CO 80309 USA; 3 Department of Electrical, Computer, and Energy Engineering, University of Colorado, Boulder, CO 80309 USA; 4 Renewable and Sustainable Energy Institute, University of Colorado, Boulder, CO 80303 USA

**Keywords:** Near-infrared spectroscopy, Imaging techniques, Sub-wavelength optics, Solar cells

## Abstract

In high fluence applications of lead halide perovskites for light-emitting diodes and lasers, multi-polaron interactions and associated Auger recombination limit the device performance. However, the relationship of the ultrafast and strongly lattice coupled carrier dynamics to nanoscale heterogeneities has remained elusive. Here, in ultrafast visible-pump infrared-probe nano-imaging of the photoinduced carrier dynamics in triple cation perovskite films, a ~20 % variation in sub-ns relaxation dynamics with spatial disorder on tens to hundreds of nanometer is resolved. We attribute the non-uniform relaxation dynamics to the heterogeneous evolution of polaron delocalization and increasing scattering time. The initial high-density excitation results in faster relaxation due to strong many-body interactions, followed by extended carrier lifetimes at lower densities. These results point towards the missing link between the optoelectronic heterogeneity and associated carrier dynamics to guide synthesis and device engineering for improved perovskites device performance.

## Abstract (web summary)

The optoelectronic performance of lead halide perovskite in highfluence applications are hindered by heterogeneous multi-polaron interactions in the nanoscale. Here, Nishda et al. spatially resolve sub-ns relaxation dynamics on the nanometer scale by ultrafast infrared pumpprobe nanoimaging.

## Introduction

**Fig. 1:**
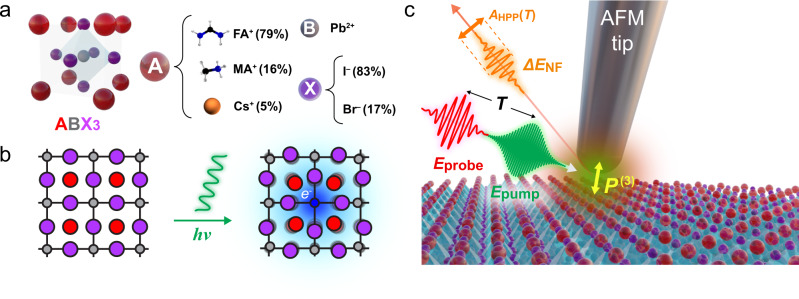
Principle of perovskite ultrafast infrared nano-imaging. **a** Unit cell structure of the triple cation perovskite $[{({{{{{{{{\rm{FA}}}}}}}}}_{0.83}{{{{{{{{\rm{MA}}}}}}}}}_{0.17})}_{0.95}{{{{{{{{\rm{Cs}}}}}}}}}_{0.05}]{{{{{{{\rm{Pb}}}}}}}}{({{{{{{{{\rm{I}}}}}}}}}_{0.83}{{{{{{{{\rm{Br}}}}}}}}}_{0.17})}_{3}$. **b** Photoinduced polaron formation in perovskites. An injected photoinduced carrier is self-trapped by the deformation of the surrounding lattice. **c** Ultrafast infrared nano-imaging of triple cation perovskite with 515 nm visible pump excitation with fluence of ~80 μJ/cm^2^, and 8.3 μm mid-infrared probe, detecting the amplitude of the transient nano-localized carrier absorption *A*_HPP_(*T*), which we attributed to either nano-localized polaron absorption or Drude carrier response.

Lead halide perovskites are organic-inorganic hybrids of the established ABX_3_ perovskite structure (Fig. 1a), with A-site cations (e.g., methylammonium MA^+^, formamidinium FA^+^ or Cs^+^) occupying the lead halide octahedral network (B = Pb^2+^, X = I^−^, Br^−^, Cl^−^). They have recently gained significant attention for a variety of optoelectronic applications, including photovoltaics^1,2^, photodetectors^3^, light-emitting diodes (LEDs), and lasers^4^. Their extraordinary photovoltaic performance, with power conversion efficiency of single-junction perovskite solar cells now exceeding 25 %^5^, has been attributed to the photo-generation of free carriers with a long lifetime, large diffusion length, and high defect tolerance^6–8^.

Strong electron-lattice coupling and large polaron formation have been proposed^9^, originating from a soft lattice exhibiting low-frequency polar fluctuations^10,11^, leading to structural deformations across multiple unit cells that stabilize the photoinduced carriers (Fig. 1b). These large polarons screen the electron-hole Coulomb interaction to enhance the carrier lifetime with modest carrier mobility^12–14^, and may account for the microscopic origin of the defect tolerance even in spin-coated polycrystalline films^15^.

Understanding the nature of the dynamic lattice deformation of perovskites has been an outstanding goal of ultrafast spectroscopy. Optical Kerr effect (OKE) spectroscopy^12,16,17^ and 2D IR spectroscopy^18–21^ have revealed lattice structural fluctuation on ~ps time scale. Slow hot carrier cooling, exceeding 10 ps, has also been demonstrated and attributed to a hot phonon bottleneck^22–24^. The dynamic evolution of polarons in perovskites has been tracked by ultrafast diffuse X-ray scattering, which resolved the lattice distortion that evolves over tens of ps^25^. Mid- to far-infrared spectroscopy proved particularly advantageous for inferring the low-energy landscape of photoinduced polaron, vibrational lattice, and coupled phonon dynamics^26,27^. Notably, in visible-pump/infrared-probe nanosecond time-resolved spectroscopy, a transient resonance assigned to a polaron absorption has recently been identified, and attributed to a transition from a phonon-trapped to free-carrier state^28–30^.

However, with these spectroscopic signals representing ensemble averages, they cannot readily provide the full pictures of the complex spatio-temporal evolution of polarons in perovskites, given the extraordinary degree of spatial heterogeneity in the optoelectronic response that ranges across atomic, domain, grain, and device scales^31–34^. To address this issue, a variety of microscopy experiments have helped resolving the spatial complexity of perovskites. Ground-state XRD microscopy has demonstrated heterogeneous lattice strain correlated with fluorescence lifetimes^35^. In the excited state, ultrafast visible-probe microscopy has probed exciton dissociation and carrier diffusion dynamics^24,36,37^, yet it lacks specificity to the evolving many-body interactions of carriers with phonons and neighboring carriers. Infrared vibrational scattering scanning near-field optical microscopy (IR *s*-SNOM) resolves nanometer spatial disorder in the cation-lattice interaction that is suggested to be associated with chemical heterogeneity^26^. While these experiments imply that lattice disorder underlies the optoelectronic non-uniformity, the key excited-state heterogeneity in electron-phonon coupling and associated polaron dynamics has remained elusive.

Here, we apply ultrafast spatio-temporal infrared nano-imaging^38–40^ to a triple cation perovskite $[{({{{{{{{{\rm{FA}}}}}}}}}_{0.83}{{{{{{{{\rm{MA}}}}}}}}}_{0.17})}_{0.95}{{{{{{{{\rm{Cs}}}}}}}}}_{0.05}]{{{{{{{\rm{Pb}}}}}}}}{({{{{{{{{\rm{I}}}}}}}}}_{0.83}{{{{{{{{\rm{Br}}}}}}}}}_{0.17})}_{3}$ (Fig. 1a) to resolve mid-infrared transient carrier absorption at the nanoscale (Fig. 1c). For injected carrier densities exceeding 10^19^ cm^−3^, and as relevant to high-fluence applications of perovskites, we observe ultrafast nano-localized infrared absorption with 10s–100s ps relaxation dynamics, depending on carrier density and associated many-body interactions. The relaxation dynamics varies spatially by 10–20% within the film both at intra- (~100 nm) and inter- (100–400 nm) grain scales. The lineshape evolution in the broadband far-field pump-probe spectra points to an increase in scattering time and associated increase in correlation length of the excited carriers. We analyze this behavior by applying both a phenomenological adiabatic polaron absorption model^30^ as well as the conventional Drude model^41^. By projecting the non-uniform relaxation dynamics into a map of polaron fit parameter evolution, the extracted polaron delocalization radii evolve from <1 nm to ~2 nm with associated increase in an effective stabilization energy from <150 cm^−1^ to ~250 cm^−1^ over a ~1 ns time scale. Equivalently, based on Drude modeling, correspondingly derived scattering times would evolve from ~2 fs to ~5 fs. We attribute the heterogeneity in the evolution of polaron delocalization to an underlying fundamental disorder in lattice dynamics and associated interaction with photoinduced carriers, which may be introduced inadvertently during synthesis or be photoinduced. These results suggest that optoelectronic properties of perovskite films may still be far from their fundamental limits and point to pathways for further improvements through the spatial and temporal control of electron-electron and electron-phonon interactions.

## Results

**Fig. 2:**
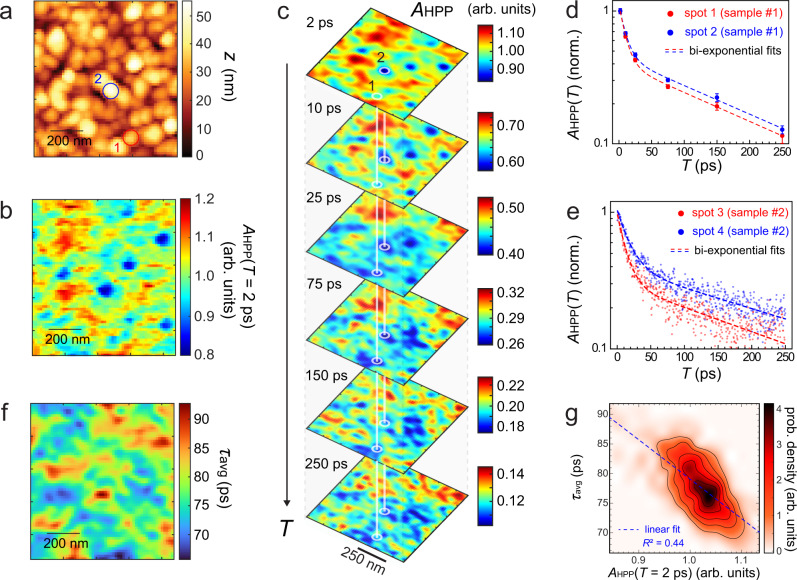
Ultrafast infrared nano-imaging of many-body carrier dynamics. AFM topography (**a**) and the corresponding heterodyne near-field pump-probe signal amplitude *A*_HPP_(*T*) image (**b**) acquired with the pump-probe delay *T* of 2 ps. **c** Time evolution of *A*_HPP_(*T*) demonstrating the heterogeneity in ultrafast carrier formation and relaxation over hundreds of nanometers. **d** *A*_HPP_(*T*) decay profiles extracted at two locations based on the imaging in **c**. **e** *A*_HPP_(*T*) decay profiles on another sample at two different locations acquired by the continuous scanning of the pump-probe delay time. **f** Image of the average decay time constant *τ*_avg_ extracted from the biexponential fits to the imaging in panel **c**. **g** Correlation plot between *A*_HPP_(*T* = 2 ps) and *τ*_avg_ (dashed, linear fit).

AFM topography (Fig. 2a) and corresponding heterodyne-detected pump-probe IR *s*-SNOM image (Fig. 2b) of the triple cation perovskite acquired at a pump-probe delay of *T* = 2 ps show the nanoscale spatial variation of the photo-induced carrier absorption *A*_HPP_(*T*). *A*_HPP_(*T*) varies by ~30% on both a grain-to-grain scale (~100 nm) as well as larger (100–400 nm) length scales. The amplitude of the *s*-SNOM signal in general is known to be highly susceptible to topographic features and may not directly be a measure of a material specific optical signature (topographic artifacts). While the variation of *A*_HPP_(*T*) is partly correlated with topography associated with the individual crystallites, a significant contribution from a topography-uncorrelated signal can be assigned to and correspondingly scales with the density of photoinduced carriers (Supplementary Fig. S3). We also show that the transient absorption nano-imaging exhibits a distinct feature from corresponding ground-state nano-imaging (Supplementary Fig. S4), which corroborates the non-trivial heterogeneity in the photoinduced carrier density (see Supporting Information for details).

The corresponding spatio-temporal evolution with varying pump-probe delays *T* (Fig. 2c) shows both a spatial and a temporal heterogeneity in the ultrafast carrier relaxation over few ps to 100s ps. The dynamics are characterized by two distinct time scales demonstrated via a biexponential fit to *τ*_1_ = 7 − 11 ps and *τ*_2_ = 150 − 220 ps, as seen from the local *A*_HPP_(*T*) evolution extracted at two representative sample locations (Fig. 2d). This range of values is typical and similar for different samples, with an example shown in Fig. 2e for two representative local *s*-SNOM traces of *A*_HPP_(*T*).

To quantify the spatial disorder in the polaron relaxation rate, we calculate the average decay time constant *τ*_avg_ derived from fits of *A*_HPP_(*T*) to biexponentials of form ${a}_{1}{e}^{-T/{\tau }_{1}}+{a}_{2}{e}^{-T/{\tau }_{2}}$ with *τ*_avg_ ≡ (*a*_1_*τ*_1_ + *a*_2_*τ*_2_)/(*a*_1_ + *a*_2_) at each sample location, yielding a nanoscale map of *τ*_avg_ (Fig. 2f). The variation in *τ*_avg_ exhibits an anti-correlation with *A*_HPP_(*T* = 2ps) (Fig. 2g), suggesting that larger polaron densities generally lead to faster relaxation (excitation induced relaxation). Correspondingly, a faster relaxation is observed with increasing pump fluence (see Supporting Information).

**Fig. 3:**
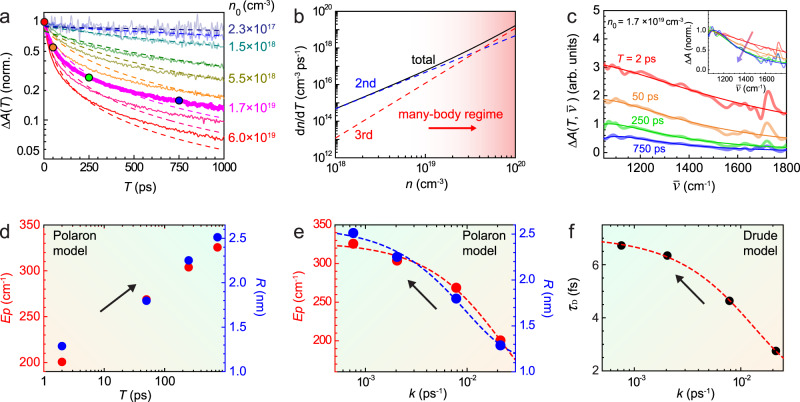
Transient broadband infrared absorption spectroscopy. **a** Transient absorption relaxation Δ*A*(*T*) for variable carrier density (solid lines), with global fit to recombination model (Eq. (1), dashed). **b** Calculated carrier relaxation rate as function of injected carrier density based on fit parameter from A, with many-body regime for densities above 10^19^ cm^−3^. **c** Transient absorption spectra ${{\Delta }}A(\overline{\nu })$ at different pump-probe delays *T* for an injected carrier density of 1.7 × 10^19^ cm^−3^. Bold line - transient absorption spectra, thin line - fitting with polaron absorption model in Eq. (2). Inset: evolution of line shape based on normalized spectra. **d** Effective polaron stabilization energy and size evolution derived from fits of spectra in C and polaron absorption in Eq. (2). See main text Eqs. (2) and (3) and associated discussion for the details of these parameters. **e** Anti-correlation between instantaneous decay rate constant *k* and effective stabilization energy *E*_*p*_ and size *R*. **f** Corresponding anti-correlation between *k* and Drude scattering time *τ*_*D*_ extracted from Drude lineshape fitting of the data in panel **c**.

In order to relate the nanoscale spatial heterogeneity in photoinduced carrier absorption to the underlying microscopic mechanisms, we perform complementary visible-pump broadband-infrared-probe transient absorption spectroscopy. Figure 3a shows the observed sub-ns evolution of the spectrally-integrated transient absorption signal Δ*A*(*T*). As can be seen, the relaxation dynamics is strongly dependent on the injected carrier density *n*_0_ calculated based on the visible pump fluence and absorption coefficient, varying from 2 × 10^17^ cm^−3^ to 6 × 10^19^ cm^−3^. This strong pump fluence dependence of the photoinduced carrier relaxation rate in perovskites, as established previously^42–45^, can be described by the following recombination model

1 $$\frac{dn}{dT}=-{k}_{2}{n}^{2}-{k}_{3}{n}^{3}$$

with time-dependent carrier density *n*, bimolecular recombination rate *k*_2_, and higher-order contribution *k*_3_. A first-order term is negligible in the high-fluence regime that we study, thus leaving only second- and third-order terms in Eq. (1)^42^. We perform a corresponding global fit (dotted lines in Fig. 3a) of all time traces for the different injected carrier densities *n*_0_, with *k*_2_ and *k*_3_ as fitting parameters. The rate constants obtained of *k*_2_ = 4.7 × 10^−10^ cm^−3^s^−1^ and *k*_3_ = 1.2 × 10^−29^ cm^−6^s^−1^ fall within the range of previously reported values for different types of perovskites and probe frequencies^42–45^. Using *k*_2_ and *k*_3_, Fig. 3b shows the derived dependence of *dn*/*dT* as a function of the injected carrier density *n*_0_, demonstrating the transition from simple bimolecular recombination at low densities to higher-order for *n*_0_ > 10^19^ cm^−3^ as discussed below.

We then spectrally resolve the transient absorption ${{\Delta }}A(T,\overline{\nu })$ signal, acquired for a constant injected carrier density of *n*_0_ = 1.7 × 10^19^ cm^−3^, but for variable pump-probe delays with results shown in Fig. 3c. We note that the spectrally narrow feature at ~1720 cm^−1^ arises from CN anti-symmetric vibration of the FA cation^20,40,46^. The narrowing of the transient absorption spectra (Fig. 3c, inset) clearly suggests that correlation lengths and scattering times associated with photoinduced carriers increase with increasing pump-probe delay. However, to the best of our knowledge, the theory of the optical response of photoinduced polarons has not yet been fully established, particularly in the high density regime aside from some studies in oxides^47,48^.

We thus first apply the simple phenomenological polaron absorption model proposed by Emin^30^. In this model, within the Franck–Condon approximation which assumes no relaxation of the lattice during the transition,the transition rate is calculated between a phonon-trapped hydrogen-like carrier wave function as a ground-state and a free-carrier state as an excited state. A larger polaron gives rise to a narrower linewidth in this model. This is because a correspondingly lower momentum would result in a momentum mismatch for the transition at higher frequencies. While the predicted absorption spectrum in this model has been suggested to deviate from that obtained by diagrammatic quantum Monte Carlo method based on Fröhlich model^49^ particularly in terms of the peak and onset energy of the absorption, the model by Emin still provides for an intuitive picture of how the polaronic delocalization of the carriers impact the linewidth of the transition. The same polaron absorption model has recently been used to interpret nanosecond time-resolved mid-infrared spectra of MAPbI_3_^28^, where a resonance at ~1200 cm^−1^ has been attributed to a polaron resonance.

The analytical form of the phenomenological polaron absorption model by Emin is given by^30^

2 $$\frac{\alpha (\omega )}{{n}_{p}}=\frac{128\pi {e}^{2}}{3m\omega c}\frac{{(\beta R)}^{3}}{{[1+{(\beta R)}^{2}]}^{4}},$$

with

3 $$\beta=\frac{\sqrt{2m(\hslash \omega -3{E}_{p})}}{\hslash },$$

with effective polaron radius *R* (corresponding to the 1/*e* distance of the exponential electron delocalization length), polaron stabilization energy *E*_*p*_, and effective electron mass *m* = 0.2*m*_*e*_^28^, and polaron density *n*_*p*_. In this model, the polaron absorption exhibits the onset of absorption at 3*E*_*p*_ (Fig. 1c) with the peak position close to 3*E*_*p*_ but with a blue-shift that depends on the polaron radius *R*. However, this absorption onset of 3*E*_*p*_ assumes the transition to a Franck–Condon state with no lattice relaxation^30^, which may not be applicable particularly for the relatively shallow phonon trapping under the high density of carriers explored in this work^50,51^. Therefore, *E*_*p*_ should only be viewed as an effective stabilization energy. The extracted values for *E*_*p*_ and *R*, as plotted in Fig. 3d, increase on ps to sub-ns time scales as shown schematically in Fig. 3e. Over the course of 1 ns, *E*_*p*_ evolves from ~200 cm^−1^ to ~350 cm^−1^ while *R* increases from ~1 to ~2.5 nm. In comparison to the lattice constant of ~1 nm^52^, this demonstrates that multiple unit cells deform collectively to stabilize the carriers at later times, justifying the fitting with large polaron absorption model^30^. We also note that the model might underestimate the polaron radius for a given linewidth by neglecting a quantum nature of the polarization field^51^.

While the polaron absorption model by Emin would reproduce the subtle resonant-like feature centered at ~1100 cm^−1^ (Fig. 3c), within the bandwidth of our mid-infrared laser source we cannot rule out a Drude response as an alternative mechanism. A Drude-like response was previously suggested in sub-microsecond time-resolved mid-infrared spectroscopy in MAPbI_3_ perovskite^53^. We thus also perform a Drude analysis with details in Supporting Information (Supplementary Fig. S10). In the case of a Drude response, the observed lineshape evolution would correspond to the Drude scattering time *τ*_D_ increasing from ~3 fs to ~7 fs over the 1 ns time window, which would signify a decrease in carrier scattering and corresponding increase in carrier mobility^41^.

By comparing the evolution of *R* and *E*_*p*_ (polaron model) in Fig. 3d and *τ*_D_ (Drude model) to the corresponding relaxation curve of Δ*A*(*T*) in Fig. 3a (bold, magenta), we find that *R*, *E*_*p*_, and *τ*_D_ are anti-correlated with respect to the instantaneous carrier relaxation rate *k*. We determine the instantaneous relaxation rate *k*, which is given by

4 $$k\equiv -\frac{1}{{{\Delta }}A(T)}\frac{d{{\Delta }}A(T)}{dT}=-\frac{1}{n(T)}\frac{dn(T)}{dT},$$

analytically from a phenomenological bi-exponential fit of Δ*A*(*T*). The combined results as shown in Fig. 3e, f then demonstrate that, based on polaron absorption, smaller and less stable polaron should lead to rapid recombination, evolving towards fewer yet more stable larger polarons. Similarly, based on the Drude analysis, the increase in carrier lifetime would correspond to longer scattering times as the carrier density decreases.

**Fig. 4:**
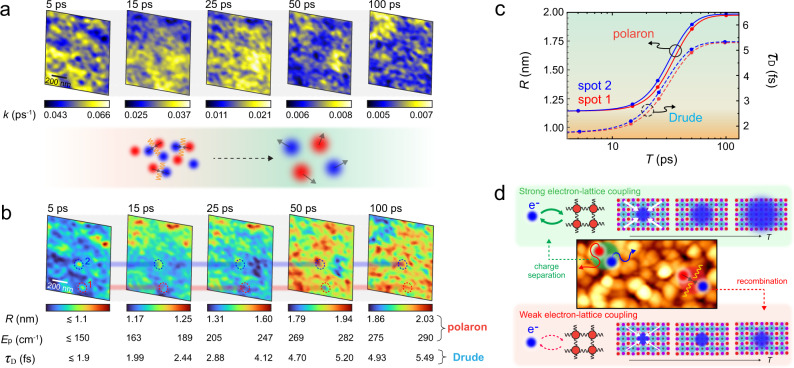
Spatio-temporal quasi-particle carrier evolution. **a** Instantaneous relaxation rate images derived from the set of spatio-temporal images of Fig. 2c via bi-exponential fitting. **b** Corresponding temporal evolution of polaron effective stabilization energy *E*_*p*_ and polaron delocalization radius *R*, or Drude scattering time *τ*_D_, based on the relationship derived in Fig. 3e, f. **c** Increase of *R*(*T*) (polaron absorption model) or *τ*_*D*_ (Drude scattering time) at two selected sample locations indicated in panel b, showing the spatial heterogeneity in the dynamic evolution of polaron size or carrier mobility. **d** Cartoon indicating the spatially non-uniform electron-lattice coupling as a potential underlying mechanism for heterogeneous carrier relaxation dynamics, which in turn results in the heterogeneity in carrier recombination and relaxation. Larger polarons associated with longer lifetime promote charge separation whereas smaller polarons with shorter lifetime are more susceptible to carrier recombination.

The anti-correlations in Fig. 3e, f allow us to relate the spatial heterogeneity in nanoscale relaxation dynamics in Fig. 2 to the underlying microscopic evolution in polaron parameters *R* and *E*_*p*_, or Drude scattering time *τ*_D_. To this end, we convert the images of the instantaneous decay rate *k* (Fig. 4a), as extracted based on bi-exponential fits of the data shown in Fig. 2b, to images of *E*_*p*_ and *R* or *τ*_D_ at each time delay with the result shown in Fig. 4b. As can be seen in the leftmost panel at early pump-probe delay times *T* < 10 ps, the extracted polaron radius *R* based on polaron absorption is significantly smaller than the perovskite lattice constant of ~1 nm. We thus only provide the upper bound of the polaron radius based on large polaron absorption model^30^. Yet for *T* > 10 ps, *R* becomes comparable to the lattice constant, and at 100 ps polarons extend across multiple unit cells.

Lastly, we compare the polaron behavior between two representative sample locations (Fig. 4c) as derived from biexponential fits to the data in Fig. 4b. As can be seen, some crystallites (spot 1, red) consistently exhibit a larger larger polaron radius *R* or scattering time *τ*_D_ than other crystallites (spot 2, blue) until they reach similar final states at a later time. Such heterogeneity suggests disorder in the interplay between electron-electron and electron-phonon interactions, which would directly impact carrier lifetimes, mobility, and thus device performance as discussed below.

## Discussion

Ultrafast infrared nano-imaging (Fig. 2) demonstrates the previously inaccessible spatial heterogeneity in carrier relaxation dynamics. With the direct probe of the photoinduced carrier absorption in the mid-infrared, we show its spatio-temporal evolution and heterogeneity on sub-ns time and sub-*μ*m length scales. This heterogeneity leading to a non-uniform photoinduced carrier density and associated nanoscale spatial heterogeneity in photon-to-carrier quantum yield is likely due to a complex interplay of varying degrees of local chemical environment, lattice strain, crystallinity, and crystallite orientations.

The observed anti-correlation between the injected polaron density *A*_HPP_(*T* = 2 ps) and relaxation time *τ*_avg_ (Fig. 2g) is characteristic of many-body interactions of carriers as expected from the high injection carrier density of *n*_0_ = 5.5 × 10^19^cm^−3^ ^22–24^. In contrast, hot carriers are unlikely to dominate the observed nano-localized mid-infrared transient absorption as follows from the observed fluence dependence where higher injected carrier densities result in faster relaxation. This behavior is not expected for the case of hot carrier dynamics, where a higher injection carrier densities would lead to slower relaxation due to the hot-phonon bottleneck effect^22–24^. We also note that the heterogeneity we observe mainly occurs at the grain-to-grain scale, in contrast to a previously observed heterogeneity of exciton segregation at individual grain boundaries^36^.

Different mechanisms have been invoked to explain transient mid-infrared resonances in semiconductors in general^54^ and perovskites in particular. However, plasma oscillation via Coulomb interaction, as established in GaAs^55,56^, can be excluded as an origin of the mid-infrared resonance we observe in the perovskite, because the peak position would red-shift as the carrier density decays, in contrast to the blue-shift we observe (Fig. 3c, d). A Rashba effect has been proposed and observed in a two-dimensional layered perovskite^57^; yet the bulk response of a three-dimensional perovskite with full inversion symmetry is unlikely to give rise to such a transition^28^.

We thus attribute our transient mid-infrared response to the optical absorption of multi-polarons. We note, however, that the nature of the optical response of such high density polarons has not yet been fully established. To phenomenologically extract the correlation lengths and times of the photoinduced carriers, we apply both the adiabatic polaron absorption model by Emin^30^ and a conventional Drude model^41^, respectively. Within the uncertainties of the experiment, the evolution of the lineshape observed can be consistently described with both models. In the case of polaron absorption^28^, the derived polaron radii evolving from *R* ~ 1 to ≤2.5 nm would be significantly smaller than the *R* ~ 10 nm previously reported for lower injected carrier density (*n*_0_ ≤ 10^17^ cm^−3^)^29^. We attribute this difference to our higher excited carrier density of *n*_0_ > 10^19^ cm^−3^, where excitation-induced many-body interactions dominate. The competition for phonon modes to couple initially blocks the formation of larger polarons (Fig. 3e) based on electron-electron interactions. The large third-order coefficient *k*_3_ = 10^−29^ − 10^−28^ cm^−6^s^−1^ in perovskites^42–45,58^ may thus be due to an effective two-body process where the binding energy is density dependent.

Our observed lineshapes are also well reproduced by the Drude model, which suggest an increase in carrier mobility due to reduced electron-electron interaction as the carrier density decays. The derived scattering times of ~7 fs at long pump-probe delays is in reasonable agreement with results from both carrier mobility^59^ and THz photoconductivity measurements^60^. Yet, given the pronounced Auger recombination in perovskites compared to conventional inorganic semiconductors, the evolving scattering time may not trivially be attributed to the simple electron-electron interactions, but includes many-body interactions also involving electron-lattice interactions with finite size effect as discussed above. With probable contributions from both models present in the multi-polaron system, it is the intricate interplay between electron-electron and electron-phonon interactions that controls the carrier relaxation dynamics in perovskites.

In the Supporting Information, we also provide the fluence-dependent transient absorption spectra that exhibit the behavior consistent with the time-dependent spectra in Fig. 3c, suggesting that the effect of hot-phonon bottleneck and other effects related to intraband relaxation in the observed signal appear to be minor (Supplementary Fig. S11). Also, based on the narrowband pump-probe measurement tracking the transient vibrational response in detail, a possible heating-induced contribution to the infrared response seems insignificant (Supplementary Fig. S12)^46^.

We attribute the spatio-temporal evolution of carrier dynamics (Fig. 4b) to the fundamental disorder in many-body interactions (Fig. 4d). The trap states with their typically lower density of *n*_trap_ ~ 10^17^ cm^−3^ ^61,62^ are unlikely to play a dominant role in inducing heterogeneity, given the much higher photo-injected carrier density of *n*_0_ = 5.5 × 10^19^ cm^−3^.

In some domains, the photoinduced carriers can be more coupled to the perovskite lattice through enhanced electron-phonon coupling. This would allow for the rapid increase in polaron size *R* and effective stabilization energy *E*_*p*_ in the polaron model. Alternatively, screened electron-electron interactions give rise to enhanced scattering time *τ*_*D*_ and enhanced mobility. In other domains, the electron-phonon coupling is less efficient, resulting in the slower growth of polarons that would compromise the stability of carriers. The spatial disorder in the perovskite lattice, previously identified by time-averaged measurements in the ground state and attributed to lattice strain^35^ and/or to chemical heterogeneity^26,63^ and associated intragrain defects^64^, likely gives rise to the observed spatial disorder in the dynamic electron-lattice interactions. We note that, due to the high-fluence excitation required to achieve sufficient contrast for nano-imaging, we cannot discern whether the heterogeneity is inherently introduced in synthesis or is photo-induced, both of which are critical for high-fluence application of perovskites.

For optoelectronic applications with high injected carrier densities (>10^17^ cm^−3^), such as LEDs, lasers, nano-photonics, or solar-concentrated photovoltaics, suppressing the density dependent higher-order many-body relaxation by eliminating domains and grains could facilitate the rapid growth of polarons as a key factor for enhancing device performance. The optimization of the electron-phonon coupling through, e.g., chemical composition^65,66^, substrate and interface control^67^, and the combination with polaritonic systems^68^, could not only help further optimize photovoltaic performance but also expand the application space of perovskite-based devices.

For photovoltaic applications with typically lower injected carrier density (~10^14^ − 10^15^ cm^−3^), the disordered electron-phonon coupling then not only becomes the source of the observed photovoltaic heterogeneity^31–34^, but may also modulate the potential landscape of carriers, resulting in non-Langevin recombination with significantly enhanced carrier lifetime^69,70^. The nanoscale heterogeneity in carrier relaxation dynamics in such a regime has recently been successfully explored with nanosecond time-resolved microwave near-field optical microscopy^71^, which attributes the heterogeneous sub-*μ*s carrier relaxation dynamics to non-uniform trap states. In contrast, in our study with the injected carrier density being much higher than the trap density, the observed heterogeneity is more sensitive to heterogeneous many-body electron-electron and electron-phonon interactions mediated by the perovskite lattice. Yet, we note that a future enhanced sensitivity and contrast of ultrafast infrared nano-imaging would also allow probing into the low-density carrier regime as, e.g., relevant for photovoltaic applications. Also, to distinguish polaron absorption and Drude response and to further elucidate the microscopic mechanism underlying the transient response, further spectroscopic extension towards the low frequency range would be necessary.

In summary, we performed ultrafast infrared nano-imaging of perovskite films, resolving a spatially heterogeneous transient infrared relaxation dynamics at ps to sub-ns time scales and nanometer sub-grain to inter-grain length scales. Far-field transient broadband infrared spectroscopy directly projects the relaxation dynamics onto the underlying spatio-temporal evolution of polaron size, stabilization energy, and Drude scattering time. The observed spatial heterogeneity and co-existence of domains with variable polaron evolution and associated carrier stabilization suggests that the device performance currently achieved based on spin-coated films is far from its fundamental limit. Equally, the current work motivates the further development of theory which can describe the photoinduced polaron absorption in the high carrier density regime with pronounced Auger recombination. The result suggests room for improvement in achieving efficient electron-phonon coupling and rapid polaron growth across a film, e.g., through further refinement in chemical composition, thin film processing, or interfacial control.

The approach of ultrafast infrared nano-imaging as demonstrated in this work is generally applicable to any type of perovskites to guide spatial engineering and nanostructuring of perovskites^72,73^ through nano-spectroscopy of polaron dynamics. This includes two-dimensional (2D) layered and quasi-2D perovskites where the control of the spatial heterogeneity in exciton dissociation and polaron formation is a key pathway to further improve their device performance^74^.

## Methods

Triple cation perovskite films of $[{({{{{{{{{\rm{FA}}}}}}}}}_{0.83}{{{{{{{{\rm{MA}}}}}}}}}_{0.17})}_{0.95}{{{{{{{{\rm{Cs}}}}}}}}}_{0.05}]{{{{{{{\rm{Pb}}}}}}}}{({{{{{{{{\rm{I}}}}}}}}}_{0.83}{{{{{{{{\rm{Br}}}}}}}}}_{0.17})}_{3}$ (Fig. 1a) are prepared by spin-coating a precursor solution onto a cleaned glass substrate^75^ for ultrafast infrared nano-imaging and onto a CaF_2_ substrate for far-field transient absorption spectroscopy. Atomic force microscopy (AFM) topography (Fig. 2a) shows grains of 100 – 200 nm in size, characteristic of fresh samples without degradation^26,76^.

We implement ultrafast heterodyne infrared (IR) nano-imaging as developed recently^40^ to probe the spatio-temporal evolution of the ultrafast carrier dynamics. As shown in Fig. 1c, 515 nm (2.4 eV) pump pulses (Pharos, Light Conversion; ~200 fs fwhm pulse duration, ~80 μJ/cm^2^ fluence, modulated by an acousto-optic modulator at Ω_M_ ~ 50 kHz) inject photoinduced carriers into the conduction band. Mid-infrared 8.3 μm pulses (Orpheus OPA/DFG, Light Conversion; ~170 fs fwhm pulse duration, ~100 μJ/cm^2^ fluence) probe the resulting carrier absorption in transient IR *s*-SNOM. The transient mid-infrared response arises from optical absorption creating a high carrier density, which we analyze applying both the phenomenological adiabatic polaron absorption model^28,30^ and the Drude model^41^ to extract correlation lengths and times of the photoninduced carrier dynamics. The repetition rate of 1 MHz ensures complete relaxation of the photoinduced carriers between subsequent laser shots. In our home-built ultrafast *s*-SNOM system, the collinear pump and probe pulses are directed to the apex of a metallic tip (Arrow-NCPt, NanoAndMore USA) in an atomic force microscope (AFM; Innova, Bruker) operating in tapping mode at the frequency *ω*_t_ ~ 250 kHz^40^.

The tip-scattered near-field signal *E*_NF_ is heterodyne amplified with a local oscillator pulse *E*_LO_ and detected by a HgCdTe detector (KLD-0.1-J1, Kolmar Technologies). The near-field pump-probe signal Δ*E*_NF_ is selectively detected by sideband lock-in detection at 2*ω*_t_ ± Ω_M_. Based on the two-phase measurement, the background-free near-field pump-probe amplitude *A*_HPP_(*T*) is isolated as a function of pump-probe delay *T*, with spatial resolution of ~50 nm limited by the radius of the tip apex^40^. Typically 1 μm × 1 μm images are acquired at 64 × 64 pixels with a scan rate of 0.2 Hz.

We avoided measuring samples with already microscopically resolvable textures, which tended to result in rapid photo-induced degradation (Supplementary Fig. S5C, D). Despite the high fluence excitation, the near-field scattering intensity (Supplementary Fig. S5A) as well as the vibrational resonance arising from the formamidinium (FA) cation (Supplementary Fig. S5B) is temporally stable^26^, suggesting minor cation migration^77^. Careful inspection of AFM topography demonstrates that, while overall topographic features are well maintained even after 1 hour of constant illumination, some domains show signatures of roughening after 20 min of illumination that has been recently attributed to photoinduced nano-grain formation of PbI_2_ (Supplementary Fig. S5E)^78^. However, PbI_2_ itself is unlikely to contribute to the observed near-field pump-probe signal due to its much weaker absorption at 2.4 eV compared with the FAMACs perovskite. We further observe that the local formation of these nano-grains does not strongly correlate with the observed relaxation dynamics in bulk domains (Supplementary Figs. S6 and S7). The two domains we analyze in detail (domain 1 and 2 in Figs. 2 and 4) do not show the features of this nano-grain formation.

We complement infrared nano-imaging by far-field visible-pump/infrared-probe transient absorption spectroscopy. To that end, a spectrally broad infrared pulse is generated by difference-frequency generation (DFG) of a 1030-nm pump with a broadband near-infrared pulse from a home-built non-collinear optical parametric amplifier (NOPA)^79^. The transmitted infrared probe pulse is subsequently detected in a symmetric Michelson interferometer. With the mid-infrared probe spanning one octave from 1000 to 1700 cm^−1^, we measure the transient absorption spectrum ${{\Delta }}A(T,\overline{\nu })$ with varying pump fluence from ~2 μJ/cm^2^ to ~150 μJ/cm^2^ at 180 kHz repetition rate.

The measurements were performed under nitrogen purge. For further details on sample preparation, ultrafast infrared nano-imaging, and far-field pump-probe transient absorption spectroscopy, see Supporting Information, Supplementary Note S1.

## Supplementary information

Supplementary Information

Peer Review File

## Data Availability

The data generated in this study have been deposited in the Open Science Framework (OSF) at https://osf.io/n8skw/.

## Code availability

The MATLAB codes developed in this study have been deposited in the Open Science Framework (OSF) at https://osf.io/n8skw/.

## References

[1] Kojima A, Teshima K, Shirai Y, Miyasaka T (2009). Organometal halide perovskites as visible-light sensitizers for photovoltaic cells. J. Am. Chem. Soc..

[2] Jena AK, Kulkarni A, Miyasaka T (2019). Halide perovskite photovoltaics: background, status, and future prospects. Chem. Rev..

[3] Huan W, Ha Kim D (2017). Perovskite-based photodetectors: materials and devices. Chem. Soc. Rev..

[4] Sutherland BR, Sargent EH (2016). Perovskite photonic sources. Nat. Photonics.

[5] Jeong J (2021). Pseudo-halide anion engineering for *α*-FAPbI3 perovskite solar cells. Nature.

[6] Lee MM, Teuscher J, Miyasaka T, Murakami TN, Snaith HJ (2012). Efficient hybrid solar cells based on meso-superstructured organometal halide perovskites. Science.

[7] Yin W-J, Shi T, Yan Y (2014). Unusual defect physics in CH_3_NH_3_PbI_3_ perovskite solar cell absorber. Appl. Phys. Lett..

[8] Kang J, Wang L-W (2017). High defect tolerance in lead halide perovskite CsPbBr_3_. J. Phys. Chem. Lett..

[9] Zhu X-Y, Podzorov V (2015). Charge carriers in hybrid organic-inorganic lead halide perovskites might be protected as large polarons. J. Phys. Chem. Lett..

[10] Yaffe O (2017). Local polar fluctuations in lead halide perovskite crystals. Phys. Rev. Lett..

[11] Franchini C, Reticcioli M, Setvin M, Diebold U (2021). Polarons in materials. Nat. Rev. Mater..

[12] Miyata K, Atallah TL, Zhu X-Y (2017). Lead halide perovskites: crystal-liquid duality, phonon glass electron crystals, and large polaron formation. Sci. Adv..

[13] Neukirch AJ (2016). Polaron stabilization by cooperative lattice distortion and cation rotations in hybrid perovskite materials. Nano Lett..

[14] Frost JM (2017). Calculating polaron mobility in halide perovskites. Phys. Rev. B.

[15] Chu W, Zheng Q, Prezhdo OV, Zhao J, Saidi WA (2020). Low-frequency lattice phonons in halide perovskites explain high defect tolerance toward electron-hole recombination. Sci. Adv..

[16] Zhu H (2016). Screening in crystalline liquids protects energetic carriers in hybrid perovskites. Science.

[17] Miyata K (2017). Large polarons in lead halide perovskites. Sci. Adv..

[18] Bakulin AA (2015). Real-time observation of organic cation reorientation in methylammonium lead iodide perovskites. J. Phys. Chem. Lett..

[19] Selig O (2017). Organic cation rotation and immobilization in pure and mixed methylammonium lead-halide perovskites. J. Am. Chem. Soc..

[20] Taylor VCA (2018). Investigating the role of the organic cation in formamidinium lead iodide perovskite using ultrafast spectroscopy. J. Phys. Chem. Lett..

[21] Nishida, J. et al. Dynamically disordered lattice in a layered Pb-I-SCN perovskite thin film probed by two-dimensional infrared spectroscopy. *J. Am. Chem. Soc.* **140**, 9882–9890 (2018). 10.1021/jacs.8b03787 30024160

[22] Yang Y (2016). Observation of a hot-phonon bottleneck in lead-iodide perovskites. Nat. Photonics.

[23] Li M (2017). Slow cooling and highly efficient extraction of hot carriers in colloidal perovskite nanocrystals. Nat. Commun..

[24] Guo Z (2017). Long-range hot-carrier transport in hybrid perovskites visualized by ultrafast microscopy. Science.

[25] Guzelturk B (2021). Visualization of dynamic polaronic strain fields in hybrid lead halide perovskites. Nat. Mater..

[26] Nishida J, Alfaifi AH, Gray TP, Shaheen SE, Raschke MB (2020). Heterogeneous cation-lattice interaction and dynamics in triple-cation perovskites revealed by infrared vibrational nanoscopy. ACS Energy Lett..

[27] Cinquanta E (2019). Ultrafast THz probe of photoinduced polarons in lead-halide perovskites. Phys. Rev. Lett..

[28] Munson KT, Kennehan ER, Doucette GS, Asbury JB (2018). Dynamic disorder dominates delocalization, transport, and recombination in halide perovskites. Chem.

[29] Munson KT, Swartzfager JR, Asbury JB (2019). Lattice anharmonicity: a double-edged sword for 3D perovskite-based optoelectronics. ACS Energy Lett..

[30] Emin D (1993). Optical properties of large and small polarons and bipolarons. Phys. Rev. B.

[31] de Quilettes DW (2015). Impact of microstructure on local carrier lifetime in perovskite solar cells. Science.

[32] Moerman D, Eperon GE, Precht JT, Ginger DS (2017). Correlating photoluminescence heterogeneity with local electronic properties in methylammonium lead tribromide perovskite thin films. Chem. Mater..

[33] Tennyson EM (2015). Nanoimaging of open-circuit voltage in photovoltaic devices. Adv. Energy Mater..

[34] Tennyson EM, Doherty TA, Stranks SD (2019). Heterogeneity at multiple length scales in halide perovskite semiconductors. Nat. Rev. Mater..

[35] Jones TW (2019). Lattice strain causes non-radiative losses in halide perovskites. Energy Environ. Sci..

[36] Nah S (2017). Spatially segregated free-carrier and exciton populations in individual lead halide perovskite grains. Nat. Photonics.

[37] Delor M, Weaver HL, Yu Q, Ginsberg NS (2019). Imaging material functionality through three-dimensional nanoscale tracking of energy flow. Nat. Mater..

[38] Sternbach AJ (2021). Programmable hyperbolic polaritons in van der Waals semiconductors. Science.

[39] Huber MA (2017). Femtosecond photo-switching of interface polaritons in black phosphorus heterostructures. Nat. Nanotechnol..

[40] Nishida J (2022). Ultrafast infrared nano-imaging of far-from-equilibrium carrier and vibrational dynamics. Nat. Commun..

[41] Yeh TT (2017). Ultrafast carrier dynamics in Ge by ultra-broadband mid-infrared probe spectroscopy. Sci. Rep..

[42] Trinh MT, Wu X, Niesner D, Zhu X-Y (2015). Many-body interactions in photo-excited lead iodide perovskite. J. Mater. Chem. A.

[43] Rehman W (2015). Charge-carrier dynamics and mobilities in formamidinium lead mixed-halide perovskites. Adv. Mater..

[44] Richter JM (2016). Enhancing photoluminescence yields in lead halide perovskites by photon recycling and light out-coupling. Nat. Commun..

[45] Wehrenfennig C, Eperon GE, Johnston MB, Snaith HJ, Herz LM (2014). High charge carrier mobilities and lifetimes in organolead trihalide perovskites. Adv. Mater..

[46] Guo P (2018). Slow thermal equilibration in methylammonium lead iodide revealed by transient mid-infrared spectroscopy. Nat. Commun..

[47] Tempere J, Devreese JT (2001). Optical absorption of an interacting many-polaron gas. Phys. Rev. B.

[48] Devreese JT, Klimin SN, Van Mechelen JL, Van Der Marel D (2010). Many-body large polaron optical conductivity in SrTi_1-x_Nb_x_O3. Phys. Rev. B.

[49] Mishchenko AS, Nagaosa N, Prokof’ev NV, Sakamoto A, Svistunov BV (2003). Optical conductivity of the Fröhlich polaron. Phys. Rev. Lett..

[50] De Filippis G, Cataudella V, Mishchenko AS, Perroni CA, Devreese JT (2006). Validity of the Franck-Condon principle in the optical spectroscopy: optical conductivity of the Fröhlich polaron. Phys. Rev. Lett..

[51] Myasnikova AE, Myasnikov EN (2008). Correlation of optical conductivity and angle-resolved photoemission spectra of strong-coupling large polarons and its display in cuprates. Phys. Rev. B.

[52] Zhang M (2017). Growth and characterization of all-inorganic lead halide perovskite semiconductor CsPbBr_3_ single crystals. CrystEngComm.

[53] Narra S, Chung C.-C., Wei-Guang Dia E, Shigeto S (2016). Simultaneous Observation of an Intraband Transition and Distinct Transient Species in the Infrared Region for Perovskite Solar Cells. J. Phys. Chem. Lett..

[54] Basov DN, Averitt RD, Van Der Marel D, Dressel M, Haule K (2011). Electrodynamics of correlated electron materials. Rev. Mod. Phys..

[55] Huber R (2001). How many-particle interactions develop after ultrafast excitation of an electron-hole plasma. Nature.

[56] Wagner M (2014). Ultrafast dynamics of surface plasmons in InAs by time-resolved infrared nanospectroscopy. Nano Lett..

[57] Zhai Y (2017). Giant Rashba splitting in 2D organic-inorganic halide perovskites measured by transient spectroscopies. Sci. Adv..

[58] Shen J-X, Zhang X, Das S, Kioupakis E, de Walle CGV (2018). Unexpectedly strong auger recombination in halide perovskites. Adv. Energy Mater..

[59] Schilcher M. J. (2021). The Significance of Polarons and Dynamic Disorder in Halide Perovskites. ACS Energy Lett..

[60] Karakus M (2015). Phonon–Electron Scattering Limits Free Charge Mobility in Methylammonium Lead Iodide Perovskites. J. Phys. Chem. Lett..

[61] Draguta S (2016). Spatially non-uniform trap state densities in solution-processed hybrid perovskite thin films. J. Phys. Chem. Lett..

[62] Kianoosh P, Timothy LK (2018). Improving the stability and decreasing the trap state density of mixed-cation perovskite solar cells through compositional engineering. Sustain. Energy Fuels.

[63] Frohna K (2021). Nanoscale chemical heterogeneity dominates the optoelectronic response of alloyed perovskite solar cells. Nat. Nanotechnol..

[64] Li W (2021). The critical role of composition-dependent intragrain planar defects in the performance of MA_1-x_FA_x_PbI_3_ perovskite solar cells. Nat. Energy.

[65] Correa-Baena JP (2019). Homogenized halides and alkali cation segregation in alloyed organic-inorganic perovskites. Science.

[66] Xu J (2020). Triple-halide wide-band gap perovskites with suppressed phase segregation for efficient tandems. Science.

[67] Chen S (2021). Stabilizing perovskite-substrate interfaces for high-performance perovskite modules. Science.

[68] Kim HS (2020). Phonon-polaritons in lead halide perovskite film hybridized with THz metamaterials. Nano Lett..

[69] Adriaenssens GJ, Arkhipov VI (1997). Non-Langevin recombination in disordered materials with random potential distributions. Solid State Commun..

[70] Munson KT, Asbury JB (2021). Influence of dynamic disorder and charge-lattice interactions on optoelectronic properties of halide perovskites. J. Phys. Chem. C..

[71] Berweger S (2022). Nanoscale photoexcited carrier dynamics in perovskites. J. Phys. Chem. Lett..

[72] Su R (2021). Perovskite semiconductors for room-temperature exciton-polaritonics. Nat. Mater..

[73] Wang K, Xing G, Song Q, Xiao S (2021). Micro- and nanostructured lead halide perovskites: from materials to integrations and devices. Adv. Mater..

[74] Blancon J-C (2017). Extremely efficient internal exciton dissociation through edge states in layered 2D perovskites. Science.

[75] Saliba M (2016). Cesium-containing triple cation perovskite solar cells: improved stability, reproducibility and high efficiency. Energy Environ. Sci..

[76] Ho K, Wei M, Sargent EH, Walker GC (2021). Grain Transformation and Degradation Mechanism of Formamidinium and Cesium Lead Iodide Perovskite under Humidity and Light. ACS Energy Lett..

[77] Mao W (2020). Light-induced reversal of ion segregation in mixed-halide perovskites. Nat. Mater..

[78] Richheimer F (2022). Ion-driven nanograin formation in early-stage degradation of tri-cation perovskite films. Nanoscale.

[79] Chen B-H, Wittmann E, Morimoto Y, Baum P, Riedle E (2019). Octave-spanning single-cycle middle-infrared generation through optical parametric amplification in LiGaS_2_. Opt. Express.

