# Supplementary material for: Nanoscale heterogeneity of ultrafast many-body carrier dynamics in triple cation perovskites
Source: Nat Commun. 2022 Nov 3;13:6582. doi: 10.1038/s41467-022-33935-0 (PMC9630529; doi:10.1038/s41467-022-33935-0)
Supplement: Supplementary file 1 — Supplementary Information [file 41467_2022_33935_MOESM1_ESM.pdf]

Supporting Information for
Nanoscale heterogeneity of ultrafast many-body carrier dynamics in triple cation perovskites

Jun Nishida,[1,2] Peter T. S. Chang,[1,2] Jiselle Y. Ye,[3] Prachi Sharma,[3] Dylan M. Wharton,[1,2] Samuel C. Johnson,[1,2] Sean E. Shaheen,[1,3,4] and Markus B. Raschke[1,2,*]

[1]*Department of Physics, University of Colorado, Boulder, CO 80309, USA*

[2]*JILA, University of Colorado, Boulder, CO 80309, USA*

[3]*Department of Electrical, Computer, and Energy Engineering, University of Colorado, Boulder, CO 80309, USA*

[4]*Renewable and Sustainable Energy Institute, University of Colorado, Boulder, CO 80303, USA*

CONTENTS

Supplementary Note S1: Experimental Method 2

Supplementary Note S2: Ultrafast Infrared Nano-Imaging 4

Supplementary Note S3: Far-field Broadband Transient Absorption Spectroscopy 12

Supplementary References 15

SUPPLEMENTARY NOTE S1: EXPERIMENTAL METHOD

Sample preparation. The triple cation perovskite films are prepared based on an established spin-coating method [1–3], with the composition of $[(FA_{0.83}MA_{0.17})_{0.95}Cs_{0.05}]Pb(I_{0.83}Br_{0.17})_3$. As a precursor solution, 18.2 mg of cesium iodide (CsI), 23.5 mg of methylammonium bromide (MABr), 77.1 mg of lead bromide ($PbBr_2$), 204.6 mg of formamidinium iodide (FAI), and 606.7 mg of lead iodide (PbI_2) are dissolved in a mixture of 800 μl of dimethylformamide (DMF) and 200 μl of N-methyl-2-pyrrolidone (NMP). The precursor solution is loaded on a pre-cleaned glass or calcium fluoride (CaF_2) substrate, and spin-coated at the rate of 4000 rpm for 10 seconds. The substrate is then soaked in an anti-solvent bath of diethyl ether for 30 seconds, followed by annealing on a hot plate at 100 °C for 10 minutes. All the synthesis procedures are performed in a nitrogen-filled glove box.

Ultrafast infrared nano-imaging. The details of our ultrafast infrared nano-imaging setup (Fig. S1A) can be found in [4] and are summarized here briefly. As a laser system, a large fraction (∼6.5 W) of a Yb:KGW regenerative amplifier laser output (Pharos, Light Conversion, wavelength $\lambda_0 = 1030$ nm, $\sim$ 200 fs fwhm pulse duration, 1 MHz repetition rate, and 7 W power) pumps an optical parametric amplifier and a difference frequency generation system (Orpheus OPA/DFG, Light Conversion) to generate tunable mid-infrared probe pulses ($\lambda_{\text{probe}} = 8000$ nm, $\sim$ 200 fs fwhm pulse duration). The minor fraction (∼0.5 W) of the Yb:KGW laser pumps a 2-mm-thick beta barium borate (BBO) crystal (Castech) to yield a visible pump pulse ($\lambda_{\text{pump}} = 515$ nm) via second harmonic generation (SHG). The pump pulse time delay is controlled by a 20-cm-long translation stage (SLLA42, SmarACT), and is modulated by an acousto-optic modulator (3110-120 442-633 NM, Gooch & Housego) at $\Omega_{\text{M}} \sim 50$ kHz.

The collinearly propagating pump and probe pulses are then steered to an asymmetric Michelson interferometer as established in conventional infrared scattering scanning near-field microscopy (IR s-SNOM) [5, 6]. In one arm of the Michelson interferometer, both pulses are focused onto the apex of a PtIr-coated tip (ARROW-NCPt, NanoAndMore USA) in an atomic force microscope (Innova AFM, Bruker) with an off-axis parabolic mirror (NA = 0.45). The scattered mid-infrared probe pulse is then interfered with a reference pulse, which is delayed in the second arm by a high precision translation stage (ANT95-50-L-MP, Aerotech, or SLC1760, SmarACT), and is detected by an HgCdTe (MCT) detector (KLD-0.1-J1, Kolmar Technologies).

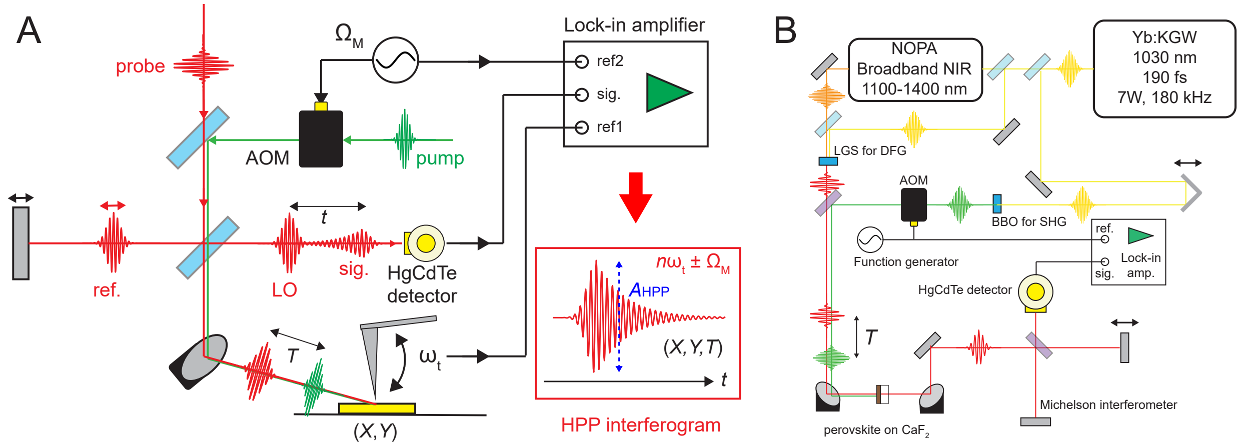

FIG. S1. **Optical Setup.** (**A**) Ultrafast infrared nano-imaging. (**B**) Far-field broadband infrared transient absorption spectroscopy.

To extract the amplitude of the nano-localized transient absorption, we employ the two-phase measurement scheme based on sideband lock-in demodulation as we established previously [4]. The AFM is operated in tapping mode with a tapping frequency $\omega_t \sim 250$ kHz, and the output from the MCT detector is then demodulated at $2\omega_t \pm \Omega_M$ based on a digital lock-in amplifier (HF2LI, Zurich Instruments). The measurements are performed with the reference pulse constructively ($\phi_{ref} = 0$) and destructively ($\phi_{ref} = \pi$) interfering with the transient nano-localized scattered field ΔE_{NF}, and the amplitude of the heterodyned near-field pump-probe signal A_{HPP} is recorded as the difference in the sideband-demodulated intensities I of the two-phase measurements, i.e., $A_{HPP} = I(\phi_{ref} = 0) - I(\phi_{ref} = \pi)$. For imaging, typically a 1 μm $\times$ 1 μm domain is scanned at a rate of 0.2 Hz to yield a 64 $\times$ 64 image. To minimize sample degradation, the AFM is enclosed and purged with nitrogen (N_2) to reduce oxygen concentration and relative humidity to less than 1 % each.

Far-field transient broadband mid-infrared absorption spectroscopy. Far-field transient broadband mid-infrared absorption spectroscopy is performed with a broadband infrared laser source based on a non-collinear parametric amplifier (NOPA) (Fig. S1B) [7]. Here, the Yb:KGW regenerative amplifier (Pharos, Light Conversion) is operated at a repetition rate of $\omega_{rep.} = 180$ kHz with total power of 7 W. ~6 W of the output pumps the NOPA to yield a broadband near-infrared pulse spanning from 1100 to 1350 nm. The difference-frequency generation is performed based on a $LiGaS_2$ (LGS) crystal between the ~0.8 W of the Yb:KGW output at 1030 nm and

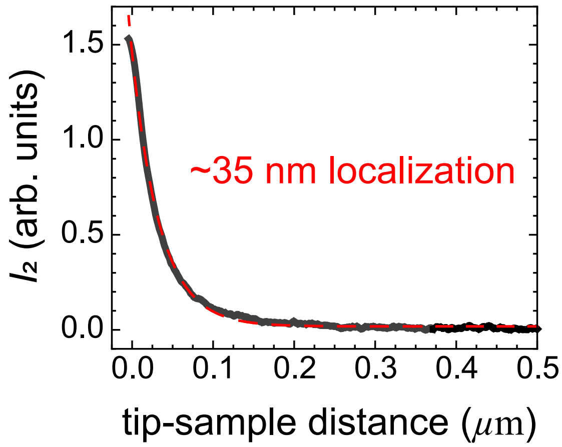

FIG. S2. **Spatial localization of near-field signal.** Tip-sample distance dependence of the near-field scattered amplitude, demonstrating the spatial localization of ~ 35 nm.

the broadband NOPA near-infrared output. The resulting mid-infrared probe pulse spectrum spans nearly one octave from 1000 to 2000 cm^{-1} (5 to 10 μm in wavelength). $\sim$0.2 W of the Yb:KGW output is then converted to a visible pump pulse via SHG based on a BBO crystal and is modulated by an AOM at $\Omega_\mathrm{M} \sim 10$ kHz.

The collinearly propagating pump and probe pulses are focused onto the triple cation perovskite film spin-coated on a CaF_2 substrate with an off-axis parabolic mirror with the focal length of $f = 100$ mm. The transmitted mid-infrared probe pulse is collimated with an identical off-axis parabolic mirror and steered into a symmetric Michelson interferometer to resolve the spectrum. The pulses from the two arms of the inteferometer are focused onto the MCT detector, and the detector output is demodulated at the laser repetition rate ($\omega_\mathrm{rep.}$) for the ground-state spectrum I, and the sideband channel ($\omega_\mathrm{rep.} \pm \Omega_\mathrm{M}$) for the excited-state spectrum ΔI. The transient absorption spectrum ΔA is then evaluated as $-\Delta I/I$, which is a good approximation with $-\Delta I/I \leq 10^{-2}$ for the visible pump fluence of ≤ 150 μJ/cm^2 used in this study.

SUPPLEMENTARY NOTE S2: ULTRAFAST INFRARED NANO-IMAGING

Spatial localization. Fig. S2 shows the tip-sample distance dependence of the self-homodyne near-field scattering intensity I_2, demonstrating <50 nm spatial localization of the tip-sample near-

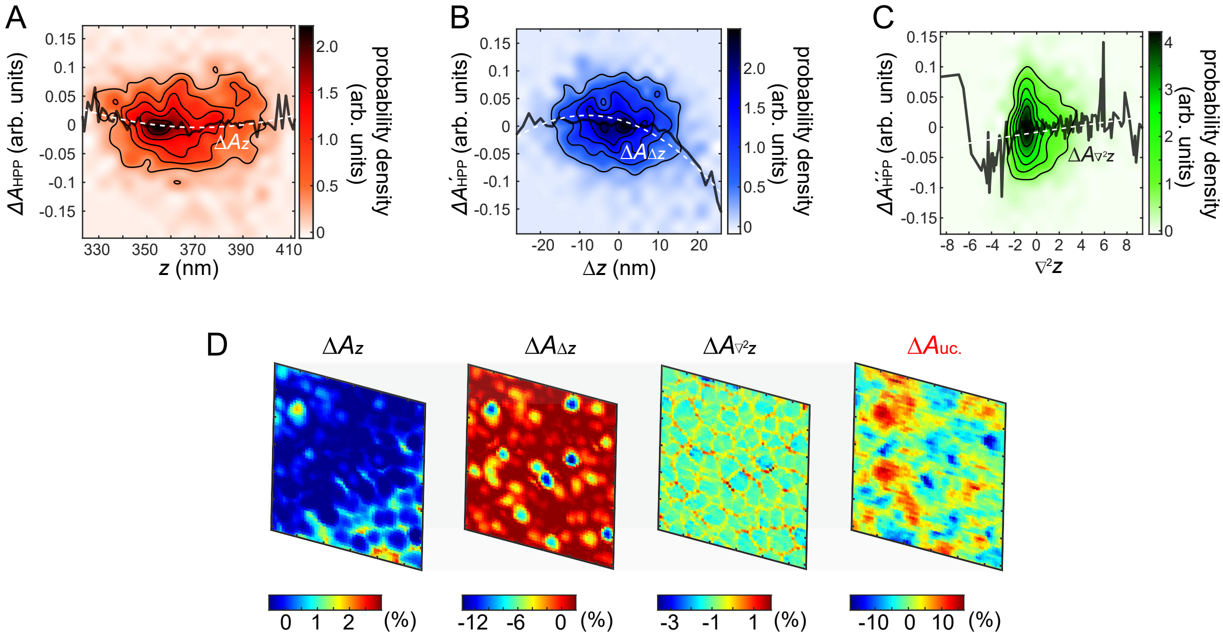

FIG. S3. **Discrimination of near-field pump-probe signal from topographic features.** **(A-C)** The correlation of A_{HPP} in Fig. 2B to the absolute height z, height variation Δz, and curvature $\nabla^2 z$, respectively. The gray line is the binned average of the y-axis value at each x axis value, while the white dotted line is the second-order polynomial fit to the binned average. **(D)** The resulting deconvolution of ΔA_{HPP} into the variations correlated to the different topographical features. See text for details.

field interaction.

Topographic artifacts. It has been established that the nano-localized scattering amplitude observed in scanning near-field optical microscopy (SNOM) can be highly susceptible to topographic feature of samples, often referred to as "topographic artifacts".

To address potential topographic artifacts in our pump-probe nano-imaging, we correlate the spatial variation of the observed nano-localized heterodyne pump-probe signal amplitude $\Delta A_{\mathrm{HPP}} = A_{\mathrm{HPP}} - \langle A_{\mathrm{HPP}} \rangle$ in Fig. 2B in the main text with the corresponding topographic features (Figure S3), with the average of the near-field pump-probe signal $\langle A_{\mathrm{HPP}} \rangle$. Fig. S3A shows the correlation with the original topography z. The gray line in Fig. S3A is the average profile of ΔA_{HPP} for each value of z, and the white dotted line is the corresponding fit to a second-order polynomial that yields the height-correlated variation ΔA_z. As seen, there is only a very weak correlation between the absolute height and the near-field pump-probe signal.

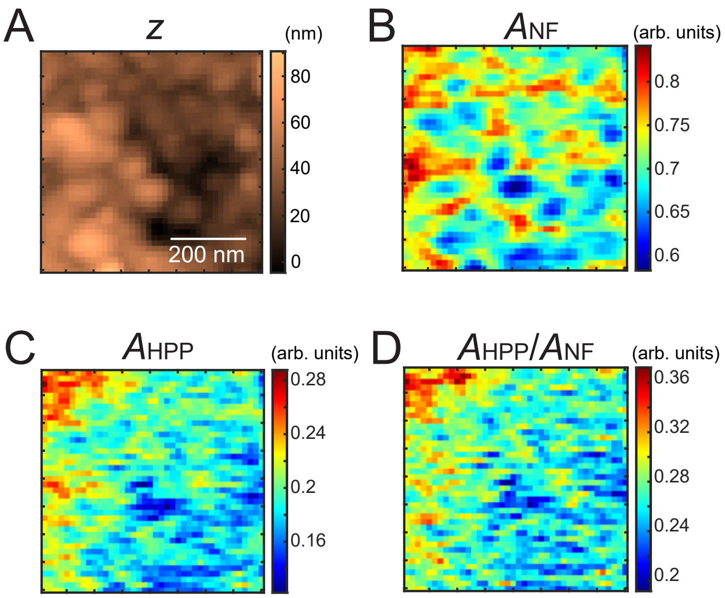

FIG. S4. **Comparison of ground-state and excited-state near-field response.** Topography (**A**), ground-state near-field scattering image $A_{\rm NF}$ demodulated at $2\omega_{\rm t}$ (**B**), and the excited-state near-field scattering image $A_{\rm HPP}(T)$ at $T = 10$ ps demodulated at $2\omega_{\rm t} \pm \Omega_{\rm M}$ (**C**). The ratio $A_{\rm HPP}(T)/A_{\rm NF}$ (**D**) exhibits a non-uniform profile, thus reflecting a non-trivial actual physical heterogeneity.

$\Delta A'_{\rm HPP} \equiv \Delta A_{\rm HPP} - \Delta A_z$ is then further correlated to the height variation Δz (Fig. S3B), defined as $\Delta z \equiv z - \langle z \rangle_{\rm tilt}$ with the overall tilt within the image $\langle z \rangle_{\rm tilt}$ provided by a 2nd order polynomial fit to the original topography. Here, a correlation with Δz is observed, with the larger values of Δz correlated with smaller $A_{\rm HPP}$. The corresponding average profile (gray line) and fit (white dotted line) give rise to $\Delta A_{\Delta z}$, the pump-probe signal variation correlated to Δz.

$\Delta A''_{\rm HPP} \equiv \Delta A'_{\rm HPP} - \Delta A_{\Delta z}$ is further correlated to the Laplacian $\nabla^2 z \equiv \partial^2 z/\partial x^2 + \partial^2 z/\partial y^2$ that quantifies the curvature at each location (Fig. S3C). The correlation $\Delta A_{\nabla^2 z}$ extracted in the identical manner is fairly weak. Finally, $\Delta A_{\rm uc.} \equiv \Delta A''_{\rm HPP} - \Delta A_{\nabla^2 z}$ yields the topographically-uncorrelated component of the near-field pump-probe signal.

Fig. S3D shows the images for each topographically correlated component, ΔA_z, $\Delta A_{\Delta z}$, $\Delta A_{\nabla^2 z}$, and $\Delta A_{\rm uc.}$, with the amplitude plotted as the fraction relative to $\langle A_{\rm HPP} \rangle$. We note that the significant variation in the near-field pump-probe signal $A_{\rm HPP}$ is *topographically uncorrelated* and can thus be attributed to the actual heterogeneity in the photoinduced carrier dynamics.

We further demonstrate a non-trivial spatial heterogeneity in the near-field pump-probe signal

$A_{\text{HPP}}(T)$, by comparing it to the ground-state near-field response A_{NF} (Fig. S4). Here, the excited-state response A_{HPP} (Fig. S4C) demonstrates a distinct profile from A_{NF} (Fig. S4B), resulting in the non-uniform profile in the ratio of the two, $\Delta A_{\text{NF}}/A_{\text{NF}}$ (Fig. S4D). We also note that the fractional variation in A_{HPP} is much larger than the variation of A_{NF}. As the underlying topography is exactly identical between A_{NF} and ΔA_{NF}, the difference cannot be attributed to a topographic artifact. This corroborates the presence of the spatial non-uniformity in the photoinduced carrier density.

Stability of the measurement. To characterize the stability of the measurement, we perform the ultrafast near-field pump-probe spectroscopy at the probe wavelength of $\sim 5.9\ \mu$m [4]. In addition to the stability in the near-field scattering level of interest, this wavelength range includes the CN anti-symmetric vibrational response from the formamidinium (FA) cation at $\sim 1715\,\text{cm}^{-1}$, allowing us to monitor the compositional stability. We continuously acquire the nano-FTIR interferograms at ~ 10 different spots, with 10-min pump illumination for each spot. In Fig. S5A, we plot the time evolution of the self-homodyne ground-state scattering intensity. During the 10 min, equivalent to the time necessary to produce one complete heterodyne pump-probe image as in Fig. 2B, the signal level on average is highly stable with only minor decay, suggesting at most minor pump-induced sample or tip alteration. In Fig. S5B, we show the stability of the vibrational signal amplitude [2, 4] arising from the CN anti-symmetric mode of the FA cation. While individual data points exhibit significant noise, on average the vibrational resonance amplitude of the FA cation does not change, suggesting at most minor change in sample composition at least from the perspective of the A-site cation. A strong pump excitation may in fact suppress pronounced ion migrations as recently suggested [8].

The stability of the sample is also corroborated by the optical image. In Fig. S5C, within the dotted area we illuminate 5 different points separated by $> 5\ \mu$m for 10 min each with the pump pulse. Yet, we cannot discern where in the sample was illuminated by the pump beam, suggesting only minor changes in the optical response in the perovskite induced by the pump pulse. However, we occasionally encounter samples which are highly susceptible to degradation (Fig. S5D). Even with the illumination time identical to that in Fig. S5C, the change in the optical profile is evident. These samples are often characterized by highly heterogeneous optical profiles even before illumination. Data from these samples were excluded from further analysis.

Regardless of the high stability of the optical signal level, over time we observe some minor

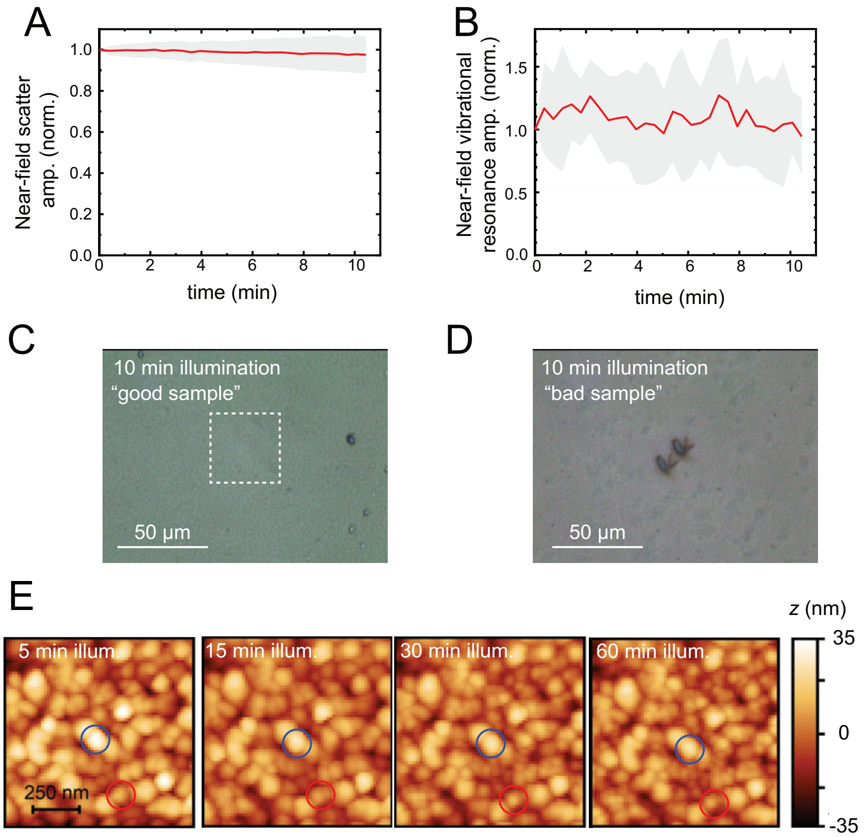

FIG. S5. **Stability of the measurement.** (A) Time evolutions of the self-homodyne near-field scattering intensity demodulated at $2\omega_t$, and (B) the amplitude of the CN anti-symmetric vibration extracted from nano-FTIR measurements. Red line - the average of the time-dependent profiles at 11 different points. Shade - 1σ standard deviation. (C) The optical image after 10 min of the pump illumination at five different points within the square dots. (D) The optical image after illumination of the pump pulse on a "bad" sample. The photoinduced degradation at the center of the image is evident. (E) The time-dependent topography under constant illumination of the pump pulse. While the overall topography is well maintained, in some domains we observe the formation of nano-grains. We analyze the grains circled by blue and red in detail in the main text.

evolution in the topographic profile in some domains (Fig. S5E). While the main grains retain their profile well, we find some domains exhibit rougher texture, which may arise from the formation of nano-grains associated with the local formation of PbI_2 as a signature of an early stage of degradation as recently suggested [9]. With the much higher bandgap of PbI_2 compared to that of the triple cation perovskite, PbI_2 is unlikely to contribute to the observed pump-probe signal. Also, by comparing Fig. S5E and Fig. 2F in the main text, we find that the local formation of the

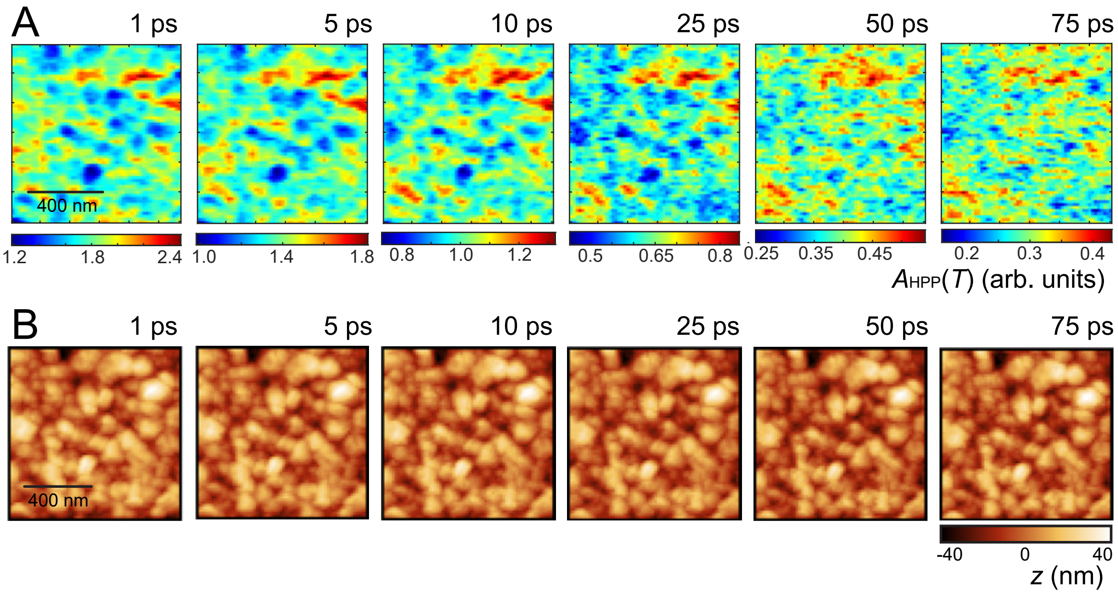

FIG. S6. **Comparison of A_{HPP} and underlying topography on another sample.** (**A**) Near-field pump-probe imaging $A_{\text{HPP}}(T)$. (**B**) Underlying topography for each pump-probe measurement.

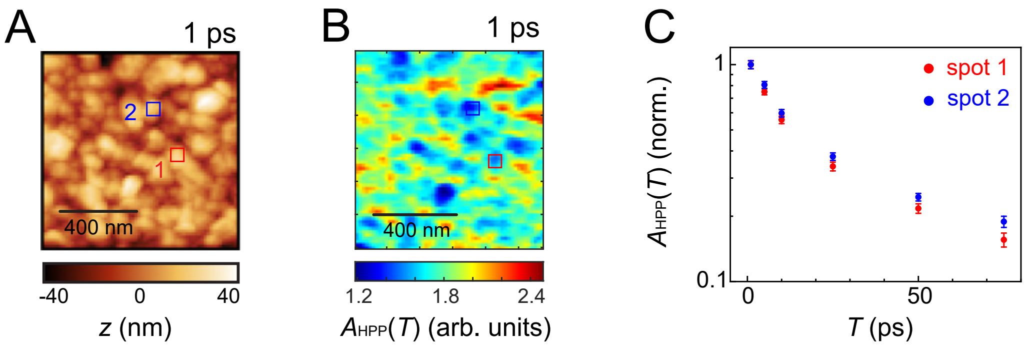

FIG. S7. **Comparing two grains with no visible roughening.** (**A**) Topography and (**B**) corresponding near-field pump-probe images from Fig. S6. (**C**) Carrier relaxation dynamics extracted from the two locations indicated.

nano-grains appears not to be correlated with the local dynamics, suggesting the photo-induced roughening is not a major source of the heterogeneity.

To further evaluate the potential impact of the photoinduced roughening to the observed relaxation dynamics, a similar measurement on another sample is performed, and the results are shown in Fig. S6. While some roughening in the topography similar to Fig. S5E is visible, its extent seems less on this sample. Some domains exhibit more pronounced roughening, yet the corre-

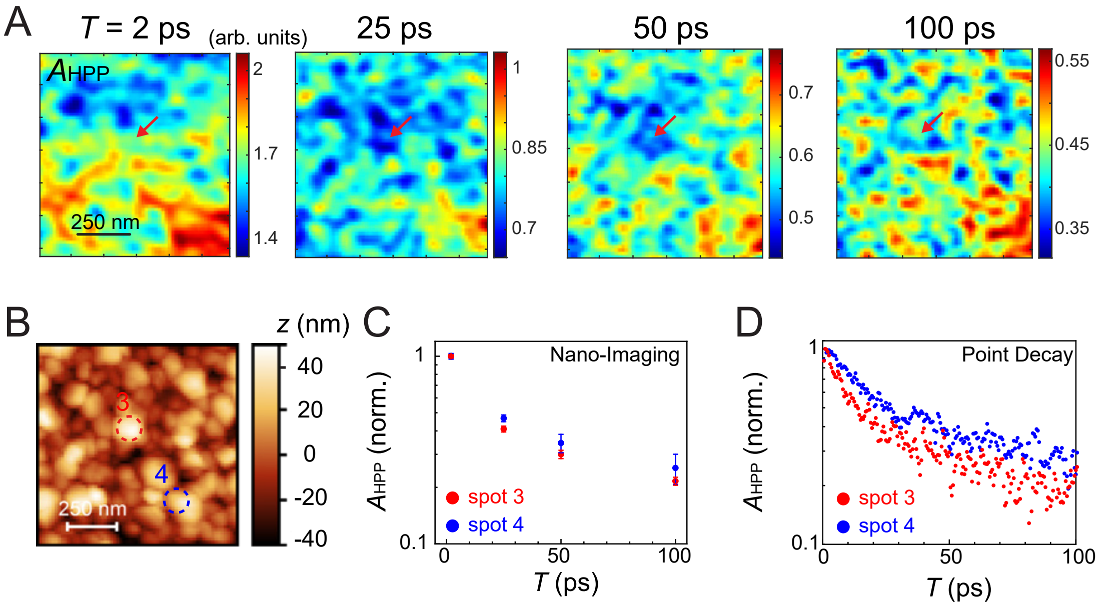

FIG. S8. **Comparison of nano-imaging to point decays.** (**A**) Pump-probe nano-imaging $A_{\mathrm{HPP}}(T)$, with a domain of a rapid decay indicated by the arrow. (**B**) Underlying topography, with spot 3 corresponding to the domain indicated by the arrow in panel A. (**C**) Carrier relaxation dynamics extracted at two spots in panel B, based on the nano-imaging. (**D**) Pump-probe decay profile at domain 3 and 4, independently measured through the point-decay measurement scheme.

sponding near-field pump-probe amplitude (ΔA_{HPP}) does not evolve in a correlated manner. In Fig. S7, we compare carrier dynamics in two domains, both of which do not exhibit the formation of the nano-grains discussed above over the course of the measurements. The dynamics are clearly distinct (Fig. S7C). These observations further corroborate that the potential photo-induced formation of nano-grains itself is not the main source of the dynamical heterogeneity that we identify. The insight might be related to a recent measurement on a similar mixed cation perovskite by nanosecond time-resolved microwave near-field microscopy, where an evolution of local photoconductivity was observed upon illumination, but the corresponding carrier relaxation dynamics was not influenced in any significant way [10].

Nano-imaging vs point decays. To ensure that the decay dynamics extracted from the pump-probe nano-imaging correctly captures the dynamical heterogeneity within the sample, we compare the decays extracted from the pump-probe nano-imaging to those from point decay measurements, i.e., by placing the tip over a domain of interest and rapidly scan the pump-probe delay.

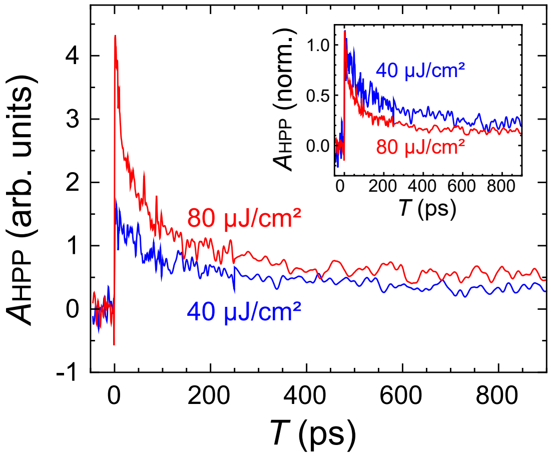

FIG. S9. **Pump fluence dependence of the near-field pump-probe relaxation.** Pump fluence dependence of the nano-localized pump-probe trace $A_{\mathrm{HPP}}(T)$ acquired at a selected and identical location within a sample. Inset - normalized profiles, demonstrating the faster decay with the higher pump fluence as consistent with the far-field measurement.

Pump-probe nano-images $A_{\mathrm{HPP}}(T)$ on yet another sample for a series of pump-probe timings are shown in Fig. S8A, together with underlying topography in Fig. S8B (We note that the slow evolution in the laser power has influenced the absolute amplitude of the A_{HPP} for this particular measurement, which is accounted for by applying time-dependent yet spatially independent factor to each pump-probe nano-imaging data). It is evident that the domain indicated by the arrows exhibit a faster decay than the surroundings, by comparing the extracted relaxation dynamics in the spots 3 and 4 (Fig. S8C; note that these are identical to the data points in Fig. 2E in the main text, noted as sample #2 spot 3 and 4). We then place the tip over spots 3 and 4 and acquired the pump-probe decays respectively (Fig. S8D). The point decay reproduces the key behavior, where the spot 3 exhibits consistently faster decay than the spot 4. These results demonstrate that the pump-probe nano-imaging accurately captures the carrier relaxation dynamics at each point.

Fluence dependence. Fig. S9 shows the fluence dependence of A_{HPP} at a selected location within a film. The relaxation with a higher pump fluence is distinctly faster than that with a lower fluence, which is consistent with our observation in the far-field pump-probe spectroscopy. This confirms that we probe bipolaron recombination dynamics in the ultrafast infrared nano-imaging as well.

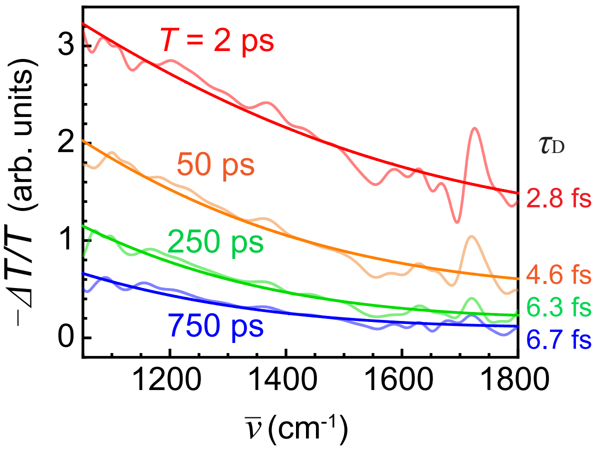

FIG. S10. **Drude model fitting.** Data identical to Fig 3C in the main text but fitting with the Drude model (dotted line), shown together with the Drude scattering time τ_D.

SUPPLEMENTARY NOTE S3: FAR-FIELD BROADBAND TRANSIENT ABSORPTION SPECTROSCOPY

Fitting with Drude model. In Fig. 3C in the main text, we fit the observed transient absorption spectra with the polaron absorption model. In Fig. S10, as alternative for comparison, we fit the same data to the Drude model. To fit to Drude model, we assume the following dielectric function $\epsilon(\omega)$ of the perovskite layer:

$$\epsilon(\omega) = \epsilon_\infty - \frac{\omega_p^2}{\omega^2 + i\omega/\tau_D}. \tag{1}$$

With no photo-doping of carriers, ϵ_∞ is set to 5, and $\omega_p = 0$ due to the absence of the free carriers. The photo-excitation renders ω_p non-zero.

We model the sample system with a four layer model, consisting of air, perovskite in the excited state, perovskite in the ground state, and the CaF_2 substrate. The total perovskite layer thickness is assumed to be 500 nm, and the pump (515 nm) penetration depth is set to 100 nm. The transfer matrix method was used to calculate the transmission with (T_{ex}) and without (T_{gnd}) the pump excitation. Then, the transient absorption is calculated as $-\Delta T/T \equiv -(T_{ex} - T_{gnd})/T_{gnd}$. ω_p and τ_D are varied as fitting parameters to reproduce the observed transient absorption spectra.

Power-dependent transient absorption spectra. Fig. S11A shows the power dependent transient absorption spectra at $T = 2$ ps, together with the fitting to Drude model. The dependence of

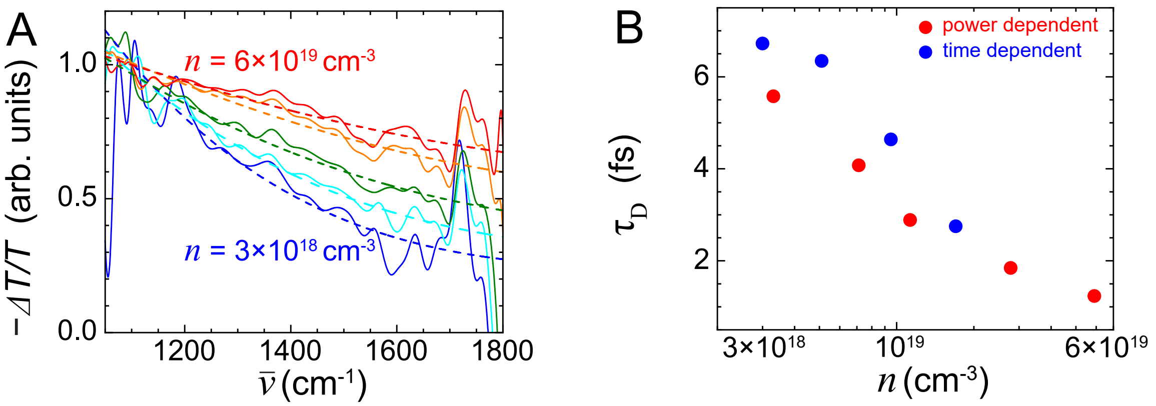

FIG. S11. **Pump power-dependent transient absorption spectra.** (**A**) Transient absorption spectra with different pump fluence varying from the injected carrier $n = 6 \times 10^{19}\,\mathrm{cm}^{-3}$ (highest) to $n = 3 \times 10^{18}\,\mathrm{cm}^{-3}$ (lowest). (**B**) Comparison of the Drude fitting parameters extracted from the power-dependent measurement in Fig. S8A and the time-dependent measurement in Fig. S7.

the lineshape on the injected carrier density is consistent with the time-dependent measurement, with the smaller instantaneous carrier density leading to faster decay toward the blue side of the lineshape.

Fig. S11B compares the Drude scattering time extracted from the power-dependent measurement in Fig. S11A and the time-dependent measurement in Fig. S10. They only differ by $< 30\%$ and are essentially identical within the uncertainty in the estimate of the instantaneous carrier density n. These observation suggests that the effects associated with hot carriers, e.g., hot phonon bottleneck, appear minor in our measurement.

Estimating possible heat-induced signal contribution. Here we discuss the pump-induced transient temperature rise, and the possible influence on the near-field signal and associated interpretation. Hot carrier thermalization on picosecond time scales or Auger recombination on hundreds of picoseconds would lead to heating. Through the temperature dependence of the mid-infrared dielectric function of the perovskite, a rise in temperature would modify the transient absorption spectrum. Yet, for the following reasons, we believe for our measurement this effect is minor.

We first estimate the single shot temperature rise. With the pump intensity of 80 μJ/cm^2 and assuming the penetration depth of $\sim$ 100 nm, the absorbed energy density is 8 J/cm^3. If all the energy turns into heat, assuming the mass density of 4 g/cm^3 [11] and the specific heat capacity of

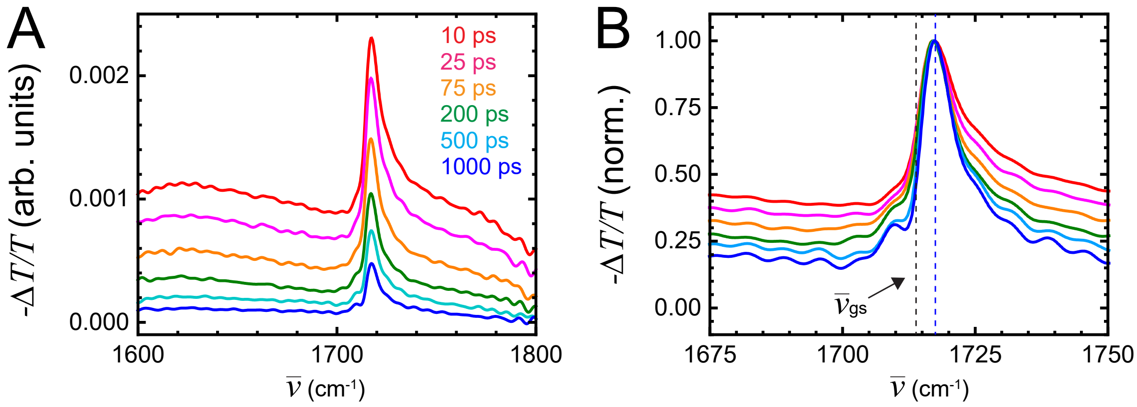

FIG. S12. **Narrow-band far-field pump-probe spectra.** (**A**) Transient absorption spectra with different pump-probe delays with resonant feature from CN anti-symmetric stretch mode of the formamidinium cation. (**B**) Normalized spectra. Black dotted line indicating the peak position for the ground-state resonance. Blue dotted line indicating the peak position for the excited-state resonance.

$308\ \mathrm{J/(kg \cdot K)}$ [12], the temperature would rise by ~ 8 K neglecting thermal diffusion.

We further use the transient vibrational CN-response as a sensitive reporter on temperature. Guo et al. [13] studied the temperature dependence of the mid-infrared transient absorption of similar perovskites. They found that the peak amplitude of the CN anti-symmetric stretch mode of the formamidinium cation in $FAPbI_3$ perovskite is highly sensitive to temperature, with a temperature increase as low as 10 K resulting in a measurable reduction of the absorptive peak amplitude. Consequently, they found that the CN anti-symmetric stretch mode appears as a reduced absorption (ground-state bleach) in the transient absorption spectra when the heat-induced contributions are dominant at long pump-probe delays.

In contrast, we consistently observe the blue-shifted positive peak in the time-dependent transient absorption spectra, as we characterized by narrow-band visible-pump infrared-probe spectroscopy (Figure S12) [2]. The ratio of the blue-shifted positive peak to the carrier-induced broadband response increases as the pump-probe timing increases, in contrast to the observation by Guo et al. [13] where they observe the growth of the ground-state bleach that overwhelms the broadband response. This shows that our transient absorption spectra are dominated by a photoinduced carrier response, with negligible thermal effects.

We also note that the it is unlikely that the heat-induced signal gives rise to a strongly frequency dependent response as we observe in Fig. 3C inset, particularly at the later pump-probe delay where a thermal effect would manifest itself most strongly.

* markus.raschke@colorado.edu

[1] Saliba, M. *et al.* Cesium-containing triple cation perovskite solar cells: improved stability, reproducibility and high efficiency. *Energy Environ. Sci.* **9**, 1989–1997 (2016).

[2] Nishida, J., Alfaifi, A. H., Gray, T. P., Shaheen, S. E. & Raschke, M. B. Heterogeneous Cation–Lattice Interaction and Dynamics in Triple-Cation Perovskites Revealed by Infrared Vibrational Nanoscopy. *ACS Energy Lett.* **5**, 1636–1643 (2020).

[3] Ye, J. Y. *et al.* Enhancing Charge Transport of 2D Perovskite Passivation Agent for Wide-Bandgap Perovskite Solar Cells Beyond 21%. *Sol. RRL* **4**, 2000082 (2020).

[4] Nishida, J. *et al.* Ultrafast infrared nano-imaging of far-from-equilibrium carrier and vibrational dynamics. *Nat. Commun.* **13**, 1–9 (2022).

[5] Huth, F. *et al.* Nano-FTIR Absorption Spectroscopy of Molecular Fingerprints at 20 nm Spatial Resolution. *Nano Lett.* **12**, 3973 (2012).

[6] Muller, E. A., Pollard, B. & Raschke, M. B. Infrared Chemical Nano-Imaging: Accessing Structure, Coupling, and Dynamics on Molecular Length Scales. *J. Phys. Chem. Lett.* **6**, 1275–1284 (2015).

[7] Chen, B.-H., Wittmann, E., Morimoto, Y., Baum, P. & Riedle, E. Octave-spanning single-cycle middle-infrared generation through optical parametric amplification in LiGaS2. *Opt. Express* **27**, 21306–21318 (2019).

[8] Mao, W. *et al.* Light-induced reversal of ion segregation in mixed-halide perovskites. *Nat. Mater.* **20**, 55–61 (2020).

[9] Richheimer, F. *et al.* Ion-driven nanograin formation in early-stage degradation of tri-cation perovskite films. *Nanoscale* **14**, 2605–2616 (2022).

[10] Berweger, S. *et al.* Nanoscale Photoexcited Carrier Dynamics in Perovskites. *J. Phys. Chem. Lett.* **13**, 2388–2395 (2022).

[11] Wei, H. & Huang, J. Halide lead perovskites for ionizing radiation detection. *Nat. Commun.* **10**, 1–12 (2019).

[12] Du, X. *et al.* Lead halide perovskite for efficient optoacoustic conversion and application toward high-resolution ultrasound imaging. *Nat. Commun.* **12**, 1–9 (2021).

[13] Guo, P. *et al.* Slow thermal equilibration in methylammonium lead iodide revealed by transient mid-infrared spectroscopy. *Nat. Commun.* **9**, 1–9 (2018).
