# Supplementary material for: Nanoscale heterogeneity of ultrafast many-body carrier dynamics in triple cation perovskites
Source: Nat Commun. 2022 Nov 3;13:6582. doi: 10.1038/s41467-022-33935-0 (PMC9630529; doi:10.1038/s41467-022-33935-0)
Supplement: Supplementary file 2 — Peer Review File [file 41467_2022_33935_MOESM2_ESM.pdf]

nature portfolio

# Peer Review File

## Title: Nanoscale heterogeneity of ultrafast many-body carrier dynamics in triple cation perovskites

**Open Access** This file is licensed under a Creative Commons Attribution 4.0 International License, which permits use, sharing, adaptation, distribution and reproduction in any medium or format, as long as you give appropriate credit to the original author(s) and the source, provide a link to the Creative Commons license, and indicate if changes were made. In the cases where the authors are anonymous, such as is the case for the reports of anonymous peer reviewers, author attribution should be to 'Anonymous Referee' followed by a clear attribution to the source work. The images or other third party material in this file are included in the article's Creative Commons license, unless indicated otherwise in a credit line to the material. If material is not included in the article's Creative Commons license and your intended use is not permitted by statutory regulation or exceeds the permitted use, you will need to obtain permission directly from the copyright holder. To view a copy of this license, visit http://creativecommons.org/licenses/by/4.0/.

**Editorial Note:** This manuscript has been previously reviewed at another journal that is not operating a transparent peer review scheme. This document only contains reviewer comments and rebuttal letters for versions considered at *Nature Communications*.

**REVIEWERS' COMMENTS**

Reviewer #2 (Remarks to the Author):

The revised version of manuscript addresses most of my concerns. The paper is sound and suitable for Nature Communications, a recommend it for publication.

Reviewer #3 (Remarks to the Author):

Authors have further provided discussion to address and clarify the concerns. By such novel experimental observation, authors present reasonable interpretation for the nanoscale heterogeneity in halide perovskite in timescale of picoseconds, which provides interesting insight into the photogenerated carrier dynamics, potentially helpful for further deep investigation of halide perovskites. I recommend accepting this manuscript to publication in Nature Communications.
